# Current Status of Blood Transfusion and Antifibrinolytic Therapy in Orthopedic Surgeries

**DOI:** 10.3389/fsurg.2015.00003

**Published:** 2015-02-12

**Authors:** Nicoleta Stoicea, Sergio D. Bergese, Wiebke Ackermann, Kenneth R. Moran, Charles Hamilton, Nicholas Joseph, Nathan Steiner, Christopher J. Barnett, Stewart Smith, Thomas J. Ellis

**Affiliations:** ^1^Department of Anesthesiology, Wexner Medical Center, Ohio State University, Columbus, OH, USA; ^2^Department of Neurological Surgery, Wexner Medical Center, Ohio State University, Columbus, OH, USA; ^3^Department of Neuroscience, Ohio State University, Columbus, OH, USA; ^4^Drexel University College of Medicine, Philadelphia, PA, USA; ^5^Temple University School of Medicine, Philadelphia, PA, USA; ^6^College of Medicine, Wexner Medical Center, Ohio State University, Columbus, OH, USA; ^7^Orthopedic One, Columbus, OH, USA

**Keywords:** blood transfusion, total hip arthroplasty, direct anterior approach (DAA), posterior approach (PA), tranexamic acid (TXA), erythropoiesis-stimulating agents (ESAs)

## Introduction

The blood loss accompanying orthopedic surgery can be significant, anemia of total hip arthroplasty being a frequent complication. Surgeons have developed different techniques for hip arthroplasty in an effort to reduce morbidity from risks such as infection, blood loss, mobility impairment, and impaired wound healing.

Since the first hip arthroplasty of the modern era was performed, Dr. Charnley’s “low-friction arthroplasty” creating the bases of two-component modern design, various surgical techniques were typically chosen according to surgeons’ personal preferences and local traditions (1). Comparisons of two common techniques, DAA and PA, did not report any conclusive data regarding the superiority of one method to the other (2–8). Among the risks of hip arthroplasty, the need for blood transfusion is a significant one. For the purpose of this review, database searches were performed using the keywords total hip arthroplasty, blood loss, transfusion, posterior approach, and direct anterior approach indicated blood transfusion as an independent risk factor for increased length of hospital stay (LOS), post-operative infections, and post-operative mobility impairment (2, 9, 10).

The newer DAA techniques are promoted as muscle-sparing and as requiring shorter recovery times (3). Although greater trochanteric intra-operative fracture and lateral femoral cutaneous nerve (LFCN) neuropraxia were more common with the DAA, all patients ultimately recovered without complications (2). Recent publications report several disadvantages associated with the DAA, the most significant being the cost of a special table (approximately $100,000) (4), the need for intra-operative fluoroscopy linked to an extended procedure length and draping contamination, LFCN injury, and bleeding from the circumflex vessels (4, 5).

An extensive retrospective analysis published by Christensen et al. in 2014 reviewed anterior and posterior THA procedures performed by a single board-certified orthopedic surgeon between 2003 and 2014. Data collected from 1,793 patients supported previous reports of increased re-intervention rates linked to the DAA (6).

Blood-management techniques in orthopedic surgeries are a ubiquitous subject within the medical literature. However, there is little consensus between anesthesiologists and surgeons on acceptable guidelines and practices (11). Commonly, efforts are made by primary care physicians (PCP), anesthesiologists, and surgeons to detect and treat anemia in patients undergoing elective orthopedic surgeries. Multiple national surveys of blood donations and transfusions within the US, found an increase of 4–5% in allogeneic blood units transfused within the last 15 years (12). Increased awareness of the disadvantages linked to allogeneic blood transfusion (ABT) following orthopedic surgeries has triggered an effort to minimizing its use. A review published by Lemaire in 2008 advised orthopedic surgeons, when performing elective surgeries, to take into consideration the preoperative erythrocyte stock, the anticipated perioperative blood loss, and the acceptable blood loss for a specific patient (13). Based on these reports, increased attention has been placed on the short-term perioperative outcome associated with different techniques used in THA. Martin et al. found that patients who underwent a DAA technique had a shorter LOS and experienced earlier mobilization compared to patients who received the PA. ABT was mentioned as a predictor of mobility failure following THA (2). A similar result was reported by Schweppe et al. while analyzing hospital-related outcomes of the DAA versus the PA in 200 patients (100 per each arm) (14). Recent prospective studies supported previous reports that the DAA results in a decreased LOS despite a longer surgical time with significantly more blood loss (15).

A review and meta-analysis of clinical outcomes including blood loss following total hip arthroplasty performed via the anterior versus posterior approach found no superiority of either method. A lower blood loss associated with the posterior approach was not considered statistically significant (16).

Given the inconsistency of existing data on recent nationwide trends in transfusion following THA, a retrospective cohort study analyzed the data collected from the US nationwide inpatient sample (NIS) for a period of 4 years between 2005 and 2008 and concluded that the incidence of blood transfusions have recently increased (17% over the past 4 years) post-operatively following THA with a great variability in practice (17).

The evidence of ABT reactions led to the development of blood-saving measures (BSMs) including preoperative erythropoietin (EPO) administration and intra-and post-operative autologous blood salvage and reinfusion. A study funded by The Netherlands Organization for Health Research and Development learned that physicians experience barriers to using BSMs due to their techniques, patient safety, and current blood-management policies. Physicians participating in the survey acknowledged using a restrictive transfusion protocol with triggers of hemoglobin threshold as low as 6.4 g/dL. However, recent trials have proved that BSMs are not cost-effective (18, 19). The combination of algorithms for blood management and restrictive transfusion thresholds contribute to surgeons’ “behavioral intention,” and whether a surgeon will tend to “watch and wait” or proceed with transfusion.

Differences of transfusion preferences amongst orthopedic surgeons may result from the necessary balance between managing the consequences of blood loss, and the risks associated with ABT (20). Even though it may be essential to support hemodynamic stability, ABT should be avoided when possible because of its associated risks, including increased rates of infection and LOS (21). A *post hoc* analysis of data pulled from the regulation of coagulation in orthopedic surgery to prevent deep-venous thrombosis and pulmonary embolism (RECORD) clinical trial program concluded that the rate of any infections and wound inflammation were higher in THA patients receiving ABT compared to patients receiving autologous blood transfusion or no transfusion (21, 22). Alteration of host T-cell regulation and microcirculatory deficits increase the risk of post-operatory infection. The overall infection rate for allogeneic recipients was documented as 9.7%. By comparison, patients receiving autologous blood transfusion had a 5.2% infection rate, which is similar to the rate of infection in patients not receiving blood at all. Furthermore, patients receiving ABT had higher rates of surgical site infection and reoperation for suspected acute peri-prosthetic infection (23).

Tranexamic acid (TXA), a synthetic derivative of the amino-acid lysine has been used to reduce blood loss and transfusion requirements in patient undergoing orthopedic surgeries based on its antifibrinolytic properties. By competitively blocking the lysine-binding sites on plasminogen, TXA is able to reduce the local degradation of fibrin by plasmin (24). Conflicting reports of increased risks of deep-vein thrombosis (DVT) in surgical patients receiving TXA led to a recent meta-analysis performed to evaluate the safety and efficacy of its use in major orthopedic surgeries. Encouraging data has shown that blood transfusion volumes per patient were significantly reduced when TXA was used, and the rate of DVT was not affected when compared with controls (25). Topical application is considered to have less systemic absorption and better local effect with the same effectiveness in reducing the blood transfusion rate as IV-TXA (26). Based on the effects of the topical form, Huang et al. published the results of a prospective study using a combination of IV and topically administered TXA in orthopedic surgery [total knee arthroplasties (TKA)]. The study found a better and faster hemostatic effect in the combined group versus the IV-TXA group, because of the higher local concentration of TXA with a smaller maximum decline of hemoglobin (27).

In 1994, Epoetin alpha was approved by the Food and Drug Administration (FDA) for the treatment of anemia associated with chronic kidney disease in order to decrease the need for transfusion. Since then several studies expressed concern over using erythropoiesis-stimulating agents (ESAs) to increase hemoglobin concentrations. These studies suggest that a rapid increase in hemoglobin concentration may trigger hemodynamic instability and serious cardiovascular events (28). Despite the potential concerns, there is data to suggest that the use of ESAs may reduce transfusion rates. A meta-analysis of 26 trials comprising 3,560 patients undergoing hip or knee arthroplasty was published in 2013. The study concluded that ESAs improved post-operative hemoglobin levels with decreased need for ABT. It is important to be aware of the safety concerns associated with ESAs when considering a more cost-effective, alternative method to ABT (29). An analysis of national trends in the utilization of blood transfusion during total hip and knee arthroplasty reported a total of 6,056,655 THA and TKAs performed in the US in the last decade, with an overall transfusion rate of 25.5% for THA and 17.9% for TKA. The records in the NIS showed that 16.4% of the THA patients and 17.9% of TKA patients received an ABT during surgery. Encouragingly, this data also supports evidence that the risk of transfusion-associated HIV and HCV has decreased dramatically within the last decade. The study encouraged the creation and utilization of a blood-management program by targeting groups of patients with a higher risk of blood transfusion in collaboration with PCP (30). A review of overall blood usage at a single, academic medical center in the Boston area found that orthopedic surgeons were frequently unsuccessful in predicting those who would require a transfusion. From a group of 62 TKA patients providing an autologous donation, only 13 required transfusions. The great majority of these autologous donations resulted in wasted resources and expense with blood draw, storage, and retrieval. The study concluded that better models are necessary to predict which patients are at an increased risk for a blood transfusion during the perioperative period. The collected data suggest that the preoperative hematocrit is the most important factor when assessing the need for blood transfusion, additional variables including age, race, gender, BMI, and comorbidities (31).

A retrospective analysis performed at Duke University Medical Center, North Carolina analyzed the influence of an increased BMI on surgeon’s decision to encourage patients to donate blood preoperatively; the “breakthrough” autologous blood transfusion was reported to decrease the infection risk when these units are received prior to any allogeneic transfusion requiring more than two units of autologous blood. The study found that BMI was not predictive of infection risk (32).

Multiple surveys addressed to Dutch and UK orthopedic surgeons within the last decade revealed an increased awareness and a positive attitude toward perioperative BSMs. The need for perioperative blood management directed toward improving patient outcome with reduced ABT is generally recognized (31, 33). The hemoglobin threshold warranting transfusion continues to be debated among anesthesiologists and orthopedic surgeons (34).

## Conclusion

As we continue to study blood-management strategies associated with hip surgery, it is important to contrast the many risk- and benefit-associated blood transfusions and the use of BSMs. More recently, the use of antifibrinolytic medication have been associated with a reduced intra-operative blood loss and the rate of post-operative transfusion. This strategy and others should be investigated further to establish their safety and effectiveness as we strive to optimize blood management during joint arthroplasty.

## Conflict of Interest Statement

The authors declare that the research was conducted in the absence of any commercial or financial relationships that could be construed as a potential conflict of interest.
